# Doxifluridine effectively kills antibiotic-resistant *Staphylococcus aureus* in chronic obstructive pulmonary disease

**DOI:** 10.1128/spectrum.01805-24

**Published:** 2024-11-12

**Authors:** Lianshen Zhang, Yingzhang Zhang, Lijie Tian, Qiang Shen, Xiaolong Ma

**Affiliations:** 1 Respiratory and Critical Care Medicine Department, Tongxiang Second People’s Hospital, Tongxiang, Zhejiang, China; 2 General Clinic, Chongfu Town Community Health Service Center, Tongxiang, Zhejiang, China; 3 Department of Respiratory, The Affiliated Hospital of Jiaxing University, Jiaxing, Zhejiang, China; Seton Hall University, South Orange, New Jersey, USA

**Keywords:** COPD, doxifluridine, MRSA, antibacterial, *G. mellonella*

## Abstract

Chronic obstructive pulmonary disease (COPD) is a leading cause of morbidity and mortality globally, often exacerbated by infections such as methicillin-resistant *Staphylococcus aureus* (MRSA). The rise in antibiotic-resistant strains complicates treatment and underscores the need for novel therapeutic drugs. In this paper, we further investigated the antimicrobial potential of a fluoropyrimidine anticancer drug doxifluridine against multidrug-resistant *S. aureus*. Determination of minimum inhibitory concentration (MIC) or minimum bactericidal concentration (MBC), monitoring of growth curve, time-kill assays, biofilm bactericidal assays, and chequerboard studies were conducted to evaluate the antibacterial efficacy of doxifluridine. Safety was assessed via hemolysis and cytotoxicity assays, and an *in vivo Galleria mellonella* larvae model was employed to test protective effects. Doxifluridine demonstrated significant antibacterial activity against clinical multidrug resistance (MDR) *S. aureus* isolates, with MIC and MBC values ranging from 0.5 to 2 µg/mL and 1 to 4 µg/mL, respectively. The results revealed doxifluridine’s potent bactericidal effects within 8 hours. Moreover, doxifluridine-treated bacteria showed a substantial reduction in biofilm mass and viability. Furthermore, chequerboard assays indicated synergistic interactions between doxifluridine and other antibiotics, reducing MIC values by two- to eightfold. More importantly, safety evaluations confirmed that doxifluridine did not exhibit hemolytic toxicity or cytotoxicity. Finally, doxifluridine significantly increased the survival rate of MRSA-infected *G. mellonella* larvae *in vivo*. In brief, doxifluridine exhibited promising *in vitro* and *in vivo* antibacterial activity against MRSA, suggesting its potential as a repurposed drug for treating resistant bacterial infections in COPD patients.

**IMPORTANCE:**

The study provides robust evidence for the antibacterial efficacy of doxifluridine against Methicillin-resistant *Staphylococcus aureus* in chronic obstructive pulmonary disease (COPD) patients. Its rapid action, ability to disrupt biofilms, and synergistic effects with other antibiotics, combined with a favorable safety profile, highlight its potential as a repurposed therapeutic agent. Future clinical trials will be essential to confirm these findings and pave the way for its integration into clinical practice. This work not only provides candidate for tackling the management of bacterial infections in COPD but also exemplifies the potential of drug repurposing in combating antibiotic-resistant infections.

## INTRODUCTION

Chronic Obstructive Pulmonary Disease (COPD) is one of the leading causes of morbidity and mortality worldwide, ranking as the fourth leading cause of death globally (1). A 2012 survey found that over 3 million patients died from COPD, accounting for 6% of all deaths globally, and this proportion has continued to increase in recent years (2). The primary causes of COPD are infectious and non-infectious factors, with infections being the predominant factor (3). COPD patients frequently experience acute exacerbations, making anti-infective therapy a crucial part of their treatment regimen. Due to the prolonged use of antibiotics, these patients are prone to various drug-resistant bacterial infections, which complicates the control of recurrent infections, exacerbates the disease, increases medical costs, and worsens the prognosis (4). Methicillin-resistant *Staphylococcus aureus* (MRSA) is a common pathogen isolated in COPD (5). COPD patients already suffer from compromised respiratory function and relatively weakened immune systems, and MRSA infections further exacerbate the disease progression (6). The multidrug resistance of MRSA significantly increases the difficulty of treatment. Therefore, the development of new drugs to control MRSA infections is crucial for the treatment of COPD.

Developing drugs to control MRSA infections is a major focus of global medical research (7). The novel antibiotics daptomycin, linezolid, and ceftaroline have been widely used to treat MRSA infections, including pneumonia and skin infections (8). Although some progress has been made, the development of MRSA-controlling drugs still faces challenges, including high research and development costs, the continuous rise of resistance, and the need for more clinical trials to validate the safety and efficacy of new drugs (9). Drug repurposing is a strategy with great potential and practical value, playing a significant role in drug development and public health (10, 11). Drug repurposing has multiple advantages, such as saving time and costs, having known safety profiles and side effects, and increasing success rates (12). The current state of drug repurposing has made significant progress in recent years (13). Metformin, originally used to treat diabetes, is now being studied for its potential in cancer prevention and anti-aging therapies (14). Chloroquine and hydroxychloroquine were widely studied for antiviral therapy during the early stages of the coronavirus disease 2019 ( pandemic, providing valuable experience for future antiviral drug research, despite the controversy over their efficacy (15). Therefore, drug repurposing is expected to offer new therapeutic options and hope for controlling MRSA infections.

Recently, studies have screened and found that the antitumor drug doxifluridine exhibits low inhibitory concentrations against vancomycin-resistant *Enterococcus faecium* (VRE) and MRSA (16, 17). As a fluoropyrimidine anticancer drug, doxifluridine has multiple cancer indications and broad clinical application prospects (18). Moreover, doxifluridine has extensive clinical usage experience and safety data, providing a reliable basis for the development of new indications and therapies (19, 20). Therefore, this study further evaluates the *in vivo* and *in vitro* antibacterial activity of doxifluridine against MRSA, aiming to provide theoretical data support for its application in clinical treatment.

## MATERIALS AND METHODS

### Bacterial strains and reagents

The clinical strains used in this study were isolated from sputum specimens of patients with COPD and were preserved in our laboratory. Strain information is detailed in Table 1. *S. aureus* ATCC29213 and MRSA ATCC43300 were obtained from the American Type Culture Collection (ATCC). The drugs used in this study were purchased from MedChemExpress. Sterile 96-well plates were obtained from Corning Inc. Bacteria were grown in Mueller-Hinton broth (MHB) or on Mueller-Hinton agar (MHA) plates at 37°C. Cells were cultured in Dulbecco’s Modified Eagle Medium (Invitrogen) containing 10% fetal bovine serum (Gibco) in an incubator set at 37°C with 5% CO_2_.

**TABLE 1:** MIC and MBC values of doxifluridine against clinically multidrug-resistant *S. aureus^a^*

Strain ID	Phenotypic properties	Source	Doxifluridine	
			MIC (μg/mL)	MBC (μg/mL)
MSSA ATCC29213			1	2
MRSA ATCC43300			2	4
*S. aureus* 181	Resistant to ME, TET, AZM, AMP, and STX	China (Zhejiang)	0.5	2
*S.aureus* 113	Resistant to TET, AZM, CN, AMP, and STX	China (Zhejiang)	0.5	1
*S.aureus* 162	Resistant to LIN, TET, AMP, CN, and LEV	China (Zhejiang)	0.5	1
*S.aureus* 169	Resistant to TET, AMP, ME, AZM, and STX	China (Zhejiang)	1	4
*S.aureus* 165	Resistant to LIN, TET, AMP, ME, and STX	China (Zhejiang)	1	4
*S.aureus* 172	Resistant to LIN, TET, AMP, LEV, and STX	China (Zhejiang)	1	4
*S.aureus* 181	Resistant to LIN, TET, ME, AMP, and LEV	China (Zhejiang)	0.5	1
*S.aureus* 185	Resistant to TET, AMP, ME, AZM, and LEV	China (Zhejiang)	2	4
*S.aureus* 176	Resistant to LIN, TET, AMP, CN, and STX	China (Zhejiang)	0.5	2
*S.aureus* 103	Resistant to TET, AMP, CN, ME, and STX	China (Zhejiang)	0.5	1

^*a*^ ME, methicillin; AMP, ampicillin; TET, tetracycline; AZM, azithromycin; CN, gentamicin; LEV, levofloxacin; LIN, lincomycin; STX, trimethoprim and sulfamethoxazole; MBC, minimum bactericidal concentration; MIC, minimum inhibitory concentration; MSSA, methicillin-susceptible *S. aureus*.

### Minimum inhibitory concentration and minimum bactericidal concentration

The minimum inhibitory concentration (MIC) of doxifluridine was determined using the broth microdilution method, following the guidelines outlined by the Clinical and Laboratory Standards Institute (21). Specifically, bacterial suspensions in the logarithmic growth phase were diluted with MHB medium to a concentration of 10^6^ colony-formingunits (CFU)/mL. Then, 50 µL of the bacterial suspension and 50 µL of doxifluridine solutions at different concentrations were added to a 96-well plate, resulting in final concentrations of 16, 8, 4, 2, 1, 0.5, 0.25, and 0.125 µg/mL. The plates were incubated at 37°C for 12–16 hours. The MIC was defined as the lowest concentration of the drug at which no visible bacterial growth was observed.

The minimum bactericidal concentration (MBC) was determined using the agar diffusion method (22). After 48 hours of incubation at 37°C, the MBC was defined as the concentration at which no bacterial colonies were observed. MIC_50_ or MBC_50_ was defined as the minimum concentration that inhibited or killed 50% of the bacterial strains, while MIC_90_ or MBC_90_ was defined as the minimum concentration that inhibited or killed 90% of the bacterial strains (23).

### Growth curve determination

The growth curve determination was performed with slight modifications as previously described (24). Bacteria in the exponential phase were diluted with MHB to a concentration of 10^6^ CFU/mL. The compounds were then added, and the mixture was transferred to 2 mL sterile eppendorf (EP) tubes. The drug concentrations were set at 0, 0.25, 0.5, 1, and 2 µg/mL. The bacterial cultures were further incubated at 37°C with shaking, and the impact of the drug on bacterial growth was assessed by measuring the absorbance at 600 nm every hour.

### Time-kill assay

The bactericidal activity curve was plotted following the method described (25). Bacteria in the exponential phase were diluted with MHB to a concentration of 10^5^ CFU/mL. The drug concentration in the bacterial suspension was set at 8 µg/mL. At 0, 2, 4, 8, 10, and 12 hours, 100 µL samples were taken and subjected to a series of five 10-fold dilutions. Each dilution (100 µL) was then placed onto sterile culture plates, inverted, and incubated at 37°C for 16–18 hours. The total number of colonies in the range of 20–300 was counted to determine the CFU average. This provided the original colony concentration in different tubes at various time points. The bactericidal activity curve was plotted using GraphPad Prism 8.0, with time points on the x-axis and the CFU per milliliter on the y-axis.

### Biofilm bactericidal assay

The biofilm bactericidal assay was conducted with slight modifications as previously described (25). *S. aureus* 43300 were suspended in a concentration of 0.2 in a volume of 100 µL and cultured in sterile flat-bottom 96-well tissue culture plates. After static incubation for 24 hours, the medium was replaced with fresh MHB. Following an additional 24 hours of incubation, the samples were washed three times with phosphate buffered saline (PBS) buffer and treated with drugs (8 µg/mL) for 12 hours. Finally, the contents of the biofilm wells were directly plated on agar plates for CFU analysis. Additionally, the crystal violet staining method was used to evaluate the efficacy of the compound in clearing bacterial biofilms (26).

### Persister assays

Based on a previous reference with minor modifications (27), cultures were grown aerobically in MHB at 37°C for 18 hours before sample grouping. Colony counts for each sample were monitored to ensure consistency, followed by the addition of antibiotics or compounds to the desired final concentration, set at 8 µg/mL. Similarly, samples were incubated aerobically at 37°C, and after 12 hours, diluted and plated on MHA for colony counting.

### Chequerboard studies

To determine the fractional inhibitory concentration index (FICI) using the checkerboard method, 50 µL of MHB medium was inoculated into each well of a 96-well plate. The two drugs were diluted along either the x-axis or y-axis. Bacteria were collected and diluted with MHB to a concentration of 1 × 10^6^ CFU/mL. Subsequently, 50 µL of the adjusted bacterial solution was added to each well. The mixture was then incubated at 37°C for 18 hours. Finally, the FIC was measured. The FICI was calculated using the formula: FICI = (MIC of drug A in combination / MIC of drug A alone) + (MIC of drug B in combination / MIC of drug B alone). The bacteriostatic interaction type was judged by FICIs as the following: FICI ≤ 0.5, synergy; 0.5 < FICI ≤ 1, additivity; and 1 < FICI < 2, indifference (or no effect); and FICI ≥2, antagonism (28).

### Cell toxicity

According to previous method (29), hemolytic toxicity of doxifluridine was determined. Four percent sheep blood cells were treated with doxifluridine (1–128 μg/mL) for 1 hour. Positive control samples were treated with 2.5% Triton X-100. The supernatant of each sample was collected, and the absorbance of the samples was measured at optical density (OD) 543. Hemolysis rate was obtained by comparing the OD 543 of each sample with the positive control.

The cytotoxicity against Vero cells was determined using the water-soluble tetrazolium salt-8 (Shanghai Targetmol) assay (30). Doxifluridine (1–128 μg/mL) and 1 × 10^5^ cells were added to a 96-well cell culture plate and cultured at 37°C for 24 hours. The cytotoxicity was then calculated by measuring the OD 450 of each sample.

### *Galleria mellonella* model

To investigate the protective effect of doxifluridine in an *in vivo* model, we utilized the established *G. mellonella* larvae model (31, 32). *G. mellonella* larvae were divided into different groups, and larvae were randomly selected (*n* = 10/group). *S. aureus* 43300 was cultured overnight and then subcultured 1:100 into fresh medium to reach mid-log phase. Bacterial cells were washed three times with PBS and diluted to OD600 = 1.0 for further use. The experimental groups included untreated (no injection), PBS (vehicle), bacterial infection, and treatment groups. Each larva was injected with 10 µL (2 × 10^8^ CFU/mL) of prepared bacteria into the right hindmost proleg. Subsequently, doxifluridine treatment (4, 8, 16, 32 mg/kg) was administered 1 hour later. Vancomycin (20 mg/kg) was used as a positive control. All drug injections were performed on the left hindmost proleg. Larvae were incubated at room temperature for 5 days, and viability counts were performed every 24 hours.

### Statistical analysis

Statistical analysis was performed using GraphPad Prism 8.0 software. All data are presented as mean ± SD. Two-tailed Student’s “*t*” test was used for statistical analysis.

## RESULTS

### Sensitivity analysis of doxifluridine against clinical MDR *S. aureus* isolates

**Fig 1:**
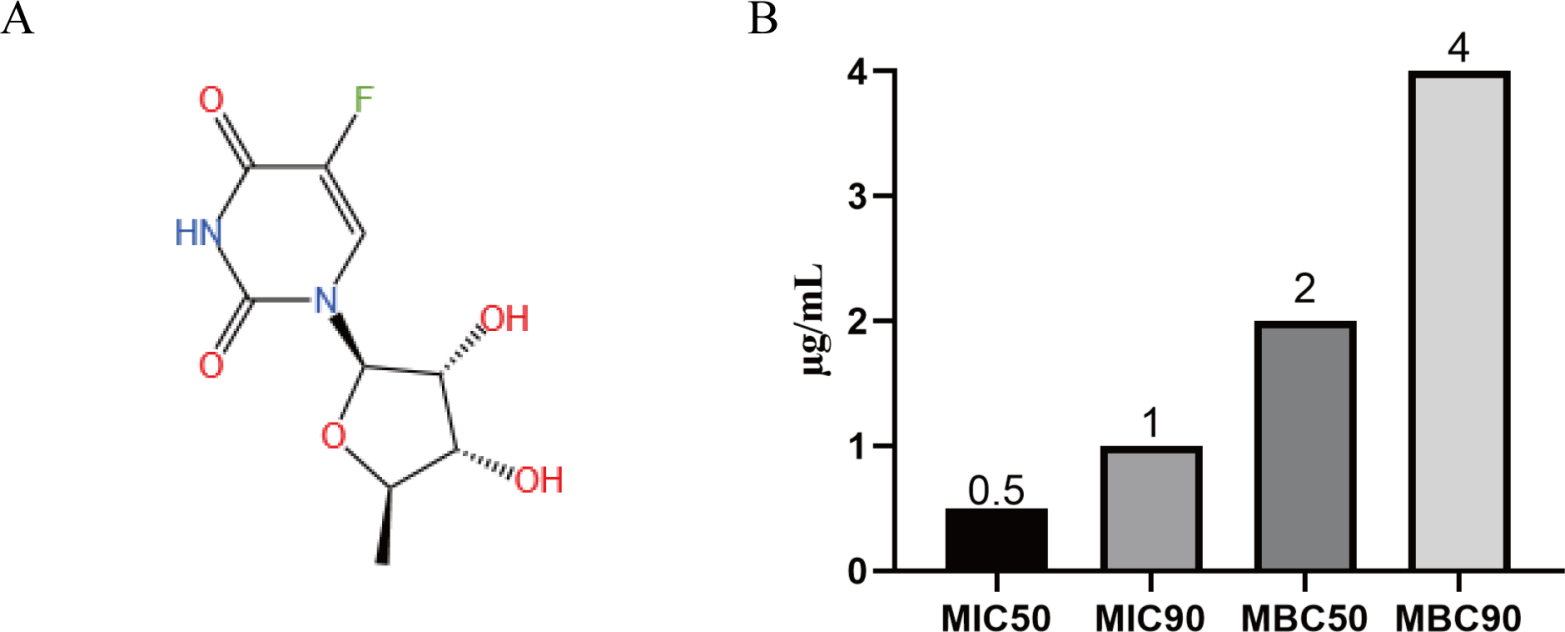
(**A**) The structure of doxifluridine; (**B**) MIC_50_ and MIC_90_ or MBC_50_ and MBC_90_ of doxifluridine against clinical MDR *S. aureus* isolates.

Previously, Younis et al. screened a library of 1,600 FDA-approved drugs and clinical molecules (clinically safe but unproven for use) to discover drugs effective against ESKAPE pathogens. Preliminary screening revealed that doxifluridine exhibited antibacterial activity against VRE and MRSA strains (17). In this study, we further evaluated the sensitivity of doxifluridine against clinical MDR *S. aureus* isolates, aiming to provide new therapeutic options and hope for controlling MRSA infections. The strains used in this study were collected from hospital laboratories, specifically from patient sputum samples containing resistant *S. aureus*. The structure of doxifluridine was shown in Fig. 1A. MIC and MBC tests revealed that doxifluridine exhibited good antibacterial activity against clinical isolates. The MIC and MBC values of doxifluridine for the tested strains ranged from 0.5 to 2 µg/mL and 1 to 4 µg/mL, respectively (Table 1). Subsequently, further analysis of the antibacterial activity of doxifluridine showed MIC_50_ and MIC_90_ values of 0.5 and 1 µg/mL, and MBC_50_ and MBC_90_ values of 2 and 4 µg/mL (Fig. 1B), respectively. These data indicated that doxifluridine may serve as a potential candidate antibiotic for the development of treatments against MDR *S. aureus*, including MRSA.

### Doxifluridine significantly inhibits the growth of methicillin-susceptible *S. aureus* (MSSA) and MRSA

**Fig 2:**
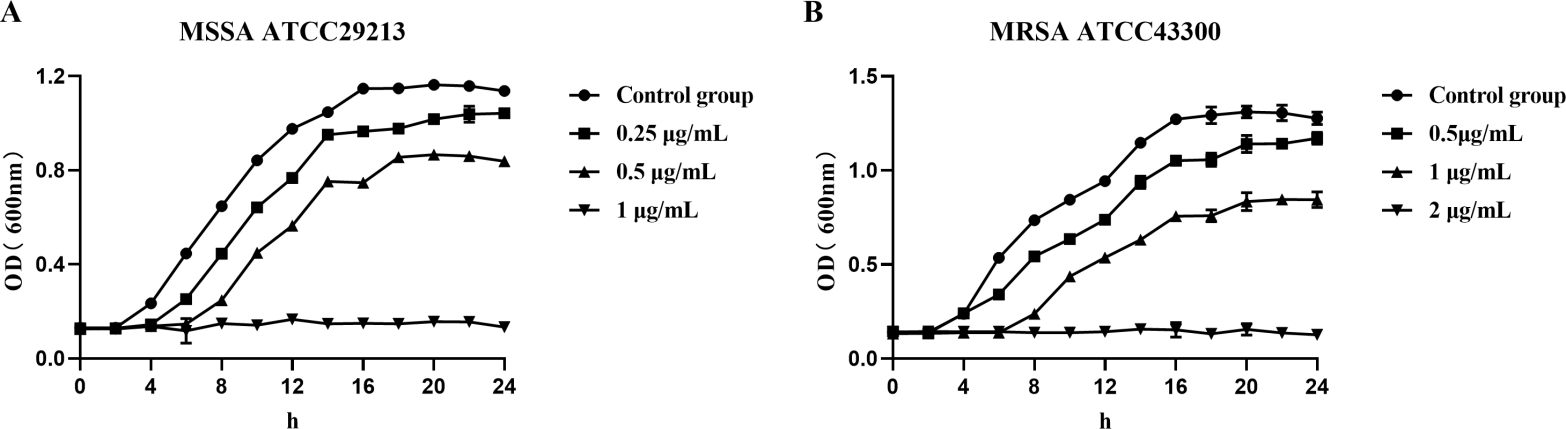
Doxifluridine significantly inhibited the growth of MSSA and MRSA. (**A**) MSSA ATCC29213; (**B**) MRSA ATCC43300. All the experiments were performed in three biological replicates. Data were presented as means ± SD.

After confirming the antibacterial activity of doxifluridine against resistant *S. aureus*, we examined the effect of this drug on the growth activity of MRSA and MSSA. Compared to the control group, treatments with 2 µg/mL and 1 µg/mL doxifluridine significantly inhibited the growth of MSSA and MRSA, with the OD values of the doxifluridine-treated groups being significantly lower than those of the control group. Moreover, doxifluridine inhibited bacterial growth in a concentration-dependent manner (Fig. 2). This further confirmed the significant inhibitory effect of doxifluridine on *S. aureus*, and the growth inhibition of MSSA and MRSA by doxifluridine at its MIC concentrations could last up to 24 hours (Fig. 2). These results indicated that doxifluridine exhibited significant antibacterial activity against both resistant and non-resistant strains.

### Antibacterial activity of doxifluridine against *S. aureus* as assessed by time-kill assay

**Fig 3:**
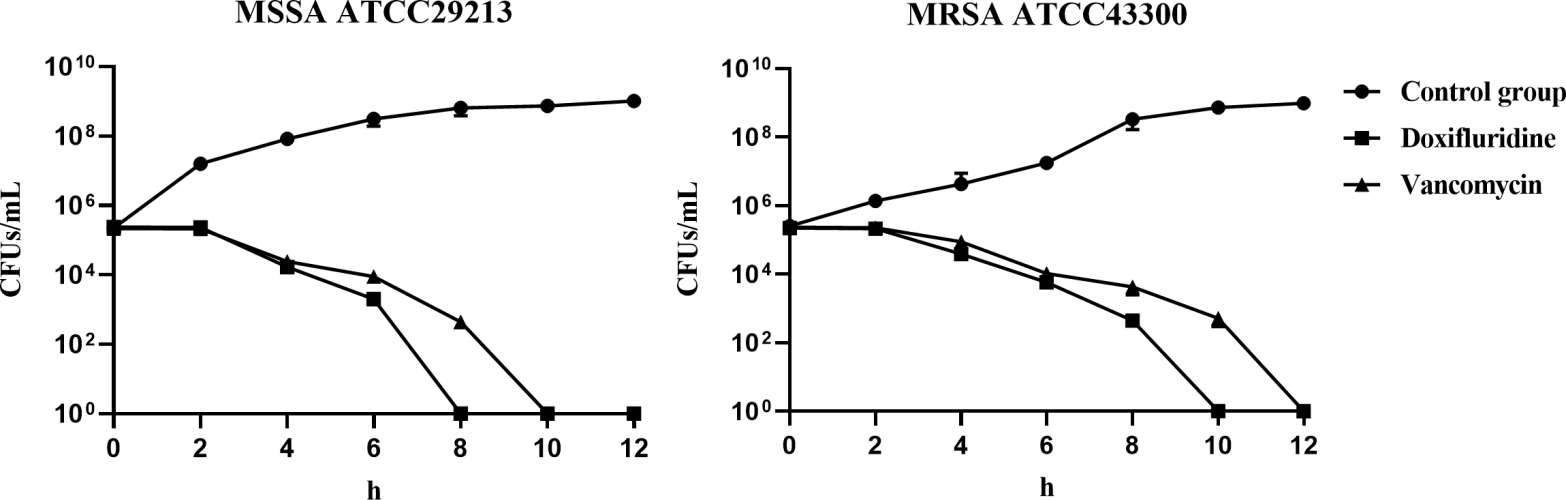
Bactericidal activity of doxifluridine against MSSA ATCC29213 and MRSA ATCC43300. The drug concentrations were all set at 8 µg/mL. All the experiments were performed in three biological replicates. Data were presented as means ± SD.

Our initial efforts focused on determining the mode of action of doxifluridine. Measuring the time required for an antibiotic to exert its bactericidal effect can provide valuable information about its mode of action. Therefore, we monitored the viability of *S. aureus* cells exposed to doxifluridine for different durations to further investigate the antibacterial mechanism of this drug. Doxifluridine at a concentration of 8 µg/mL killed nearly all MSSA ATCC29213 and MRSA ATCC43300 within 8 or 10 hours, while vancomycin killed nearly all MSSA ATCC29213 and MRSA ATCC43300 within 10 or 12 hours (Fig. 3). These results suggested that doxifluridine exhibited more potent bactericidal activity against MRSA and MSSA compared to vancomycin *in vitro*.

### Effect of doxifluridine on *S. aureus* biofilm

**Fig 4:**
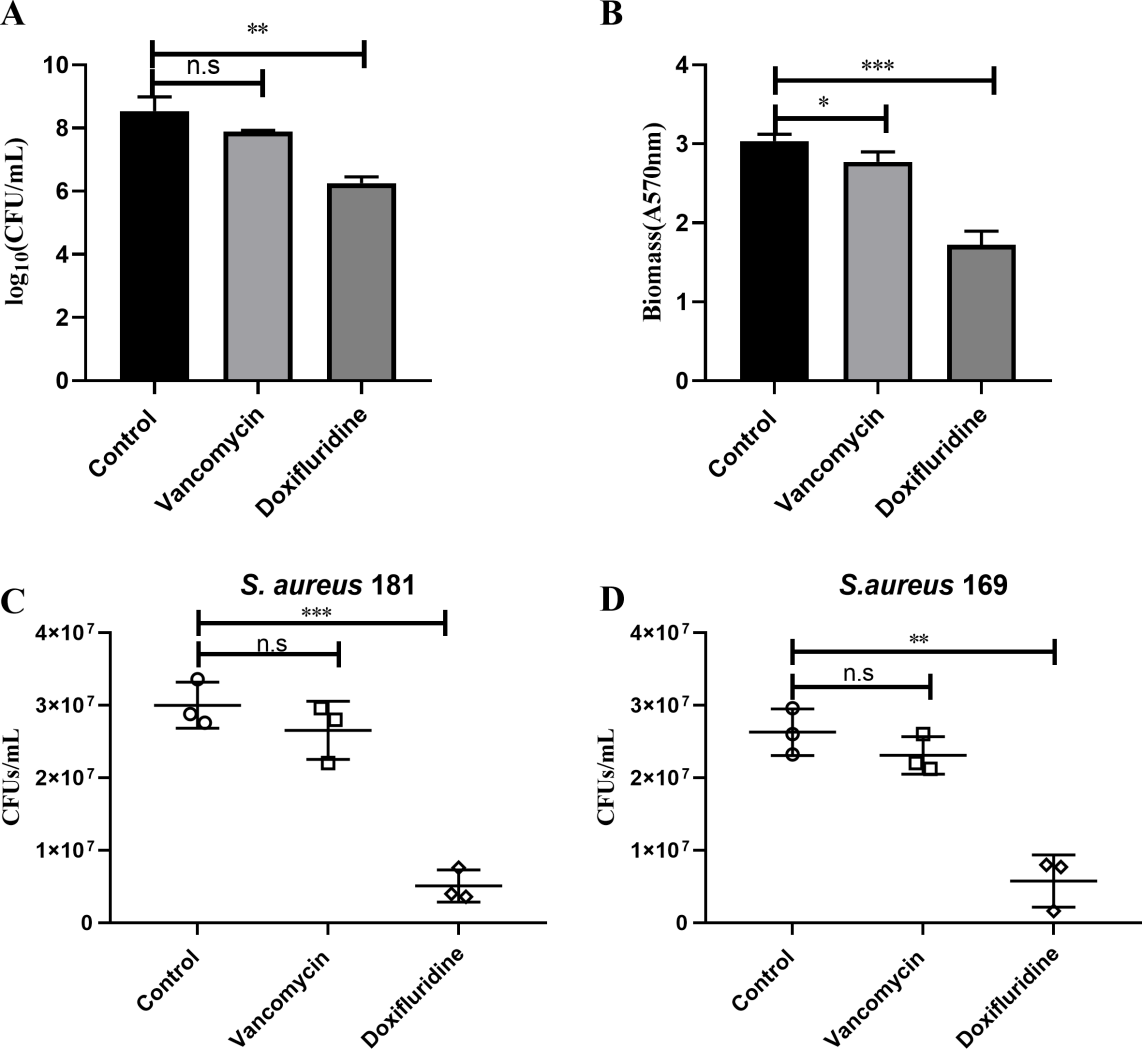
The clearance effect of doxifluridine on the mature biofilm of MRSA ATCC43300 strain. (**A**) Drugs reduce the number of viable bacteria in biofilms.The concentrations of both doxifluridine and vancomycin were set to 8 µg/mL; (**B**) effect of drugs on the mature biofilm of bacteria detected by the crystalline violet method; doxifluridine exhibited bactericidal activity against persister cells of multidrug-resistant *S. aureus* 181 (C) or 169 (**D**). All data were obtained from three biological replicates, and the mean ± SD was shown. Two-tailed Student’s “*t*” test was used for statistical analysis. (n.s, no significance; *P < 0.1, **P < 0.01, ***P < 0.001).

Bacterial biofilms are organized bacterial communities that adhere to biotic or abiotic surfaces, secreting a polymeric matrix that encapsulates them (33). Biofilm formation plays an important role in the process of *S. aureus* resisting antibiotic killing and evading clearance by the host immune system, leading to recurrent infections and antibiotic treatment failures in clinical settings (34). The data on the viable bacterial count in biofilms also showed that, compared to the blank control group and the vancomycin control group, doxifluridine treatment reduced the bacterial count by 2.28 and 1.63 log units, respectively (Fig. 4A). Similarly, crystal violet staining showed that the biofilm clearance rate was 43% at a doxifluridine concentration of 8 µg/mL, and the biofilm clearance rate of vancomycin was only 9% under the same concentration condition (Fig. 4B). Additionally, doxifluridine could reduce the number of persister cells in multidrug-resistant *S. aureus*, with a reduction of 0.6 to 0.8 log compared to the control group (Fig. 4C and D). Therefore, doxifluridine exhibited significant clearance effects on mature *S. aureus* biofilms.

### Enhancement of antibiotic activity by doxifluridine

**Fig 5:**
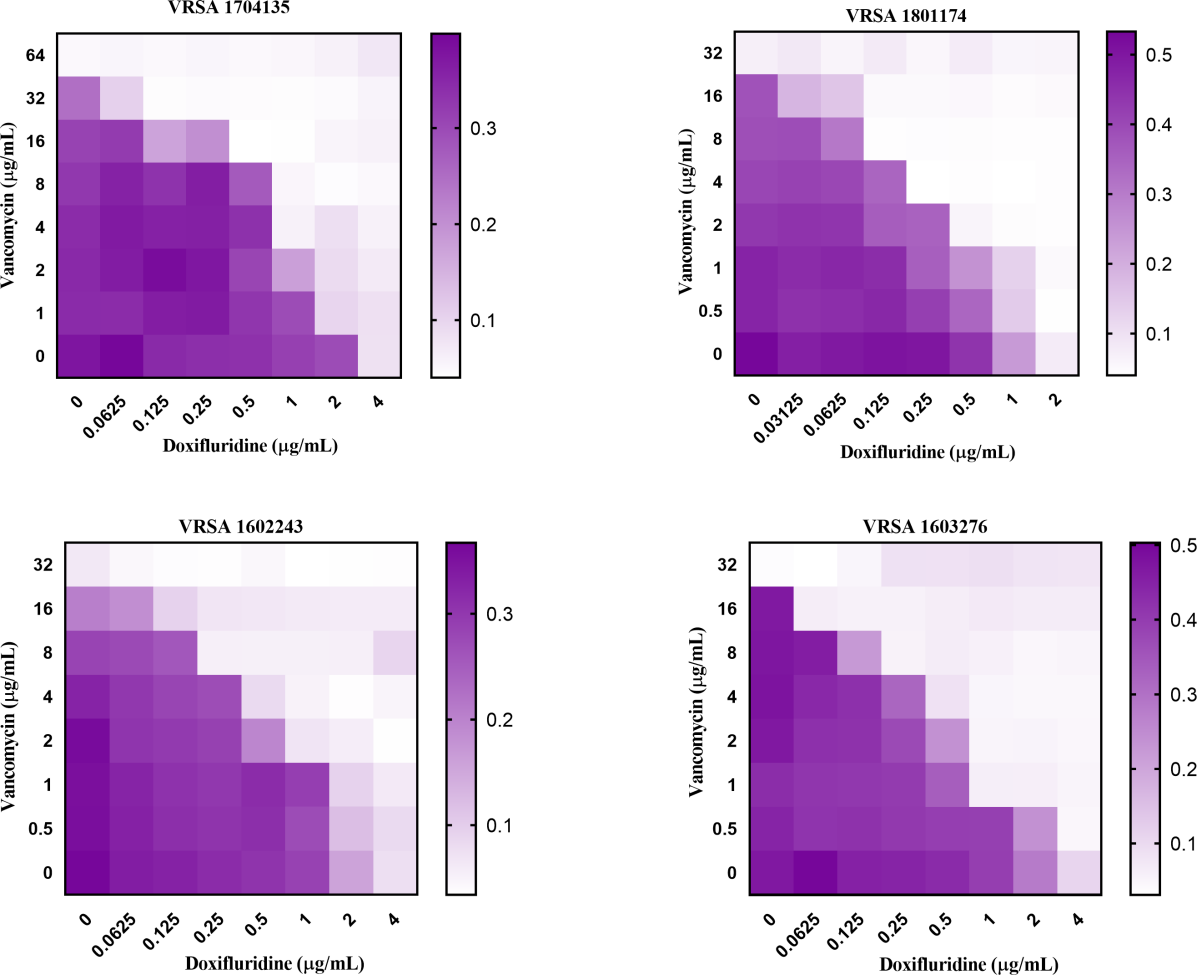
Microdilution checkerboard analysis demonstrated the combined effect of doxifluridine and vancomycin against vancomycin-resistant *S. aureus* (VRSA) strains (1704135, 1801174, 1602243, and 1603276, respectively).

The clinical treatment of MRSA infections often employs combination therapy. Combination therapy can enhance the efficacy against *S. aureus* infections, especially for methicillin-resistant strains, and provide broader bacterial spectrum coverage, particularly for unknown or complex infections (35). Therefore, the combined effects of doxifluridine and different antibiotics were tested on clinical MRSA isolates, standard MRSA strains, and standard MSSA strains. Doxifluridine significantly reduced the MIC of antibiotics (tetracycline, ampicillin, azithromycin, and methicillin) against clinically resistant strains of *S. aureus*. The effects of the drug combinations are shown in the table. The FICI of doxifluridine combined with antibiotics against the three *S*. *aureus* strains ranged from 0.25 to 0.75 (Table 2). The MIC values for all tested strains decreased by two- to eightfold when treated with doxifluridine (Table 2). Furthermore, using isolated vancomycin-resistant strains, this study evaluated the synergistic interaction between doxifluridine and vancomycin, revealing a synergistic effect with a FICI index of less than 0.5 (Fig. 5). These results suggested that doxifluridine could effectively inhibit the growth of *S. aureus* when used in combination with antibiotics.

**TABLE 2:** The FICI of doxifluridine with antibiotics in tested bacterial strains

Strain ID	Antibiotic	MIC (μg/mL)		
		Alone	Combination	FIC index
MSSA ATCC29213	Tetracycline	0.5	0.125	0.5
	Ampicillin	1	0.125	0.25
	Azithromycin	1	0.5	0.75
	Methicillin	1	0.125	0.25
MRSA ATCC43300	Tetracycline	16	2	0.375
	Ampicillin	64	8	0.25
	Azithromycin	16	2	0.25
	Methicillin	16	2	0.3125
*S. aureus* 181	Tetracycline	32	8	0.75
	Ampicillin	16	2	0.25
	Azithromycin	16	4	0.5
	Methicillin	32	4	0.25
*S.aureus* 169	Tetracycline	64	16	0.5
	Ampicillin	32	2	0.3125
	Azithromycin	16	4	0.375
	Methicillin	32	8	0.75
*S.aureus* 185	Tetracycline	32	4	0.625
	Ampicillin	32	4	0.25
	Azithromycin	16	4	0.5
	Methicillin	16	4	0.75

### Safety evaluation of doxifluridine

**Fig 6:**
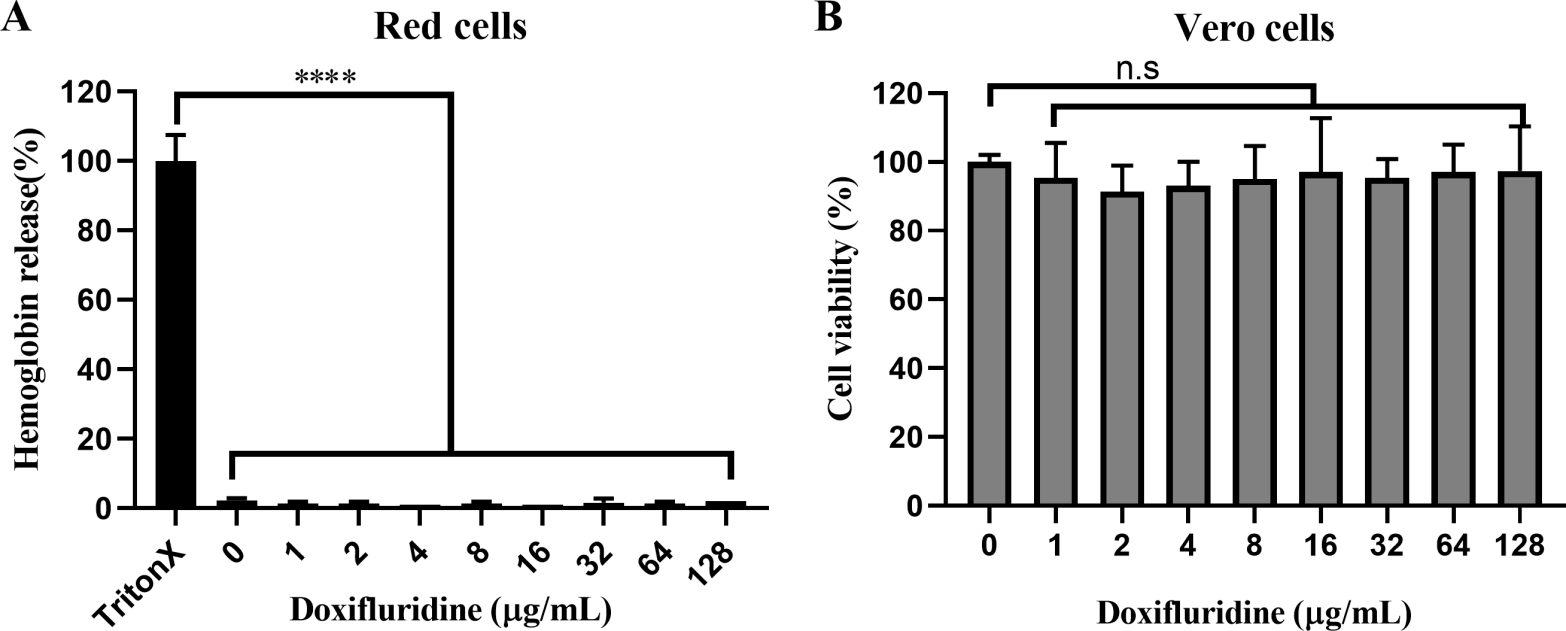
The toxicity assessment of doxifluridine *in vitro*. (**A**) The hemolytic effect of doxifluridine on red blood cells; (**B**) the cytotoxicity of doxifluridine on Vero cells. All the experiments were performed in three biological replicates. Data were presented as means ± SD. (n.s, no significance; ****P < 0.0001)

Safety evaluation is a critical aspect of drug application, and a key factor limiting the clinical use of a drug is its potential toxicity. Old drugs have undergone extensive clinical trials and long-term use, accumulating a wealth of data on their safety and toxicity (12). Doxifluridine, which possesses anticancer activity, is a prodrug of 5-fluorouracil (5-FU) (36). Despite numerous studies reporting on the toxicological information of doxifluridine (37, 38), we further assessed the *in vitro* cytotoxicity of doxifluridine in this study. Hemolysis assays demonstrated that doxifluridine did not induce hemolysis of mammalian red blood cells at concentrations ranging from 1 to 128 µg/mL (Fig. 6A). Moreover, in the Vero cell line, doxifluridine exhibited negligible cytotoxic effects (Fig. 6B). Overall, these results suggested that further research into doxifluridine as a potential drug for controlling MRSA infections was warranted, with potential for future clinical application.

### Doxifluridine protected *G. mellonella* larvae from MRSA infection

**Fig 7:**
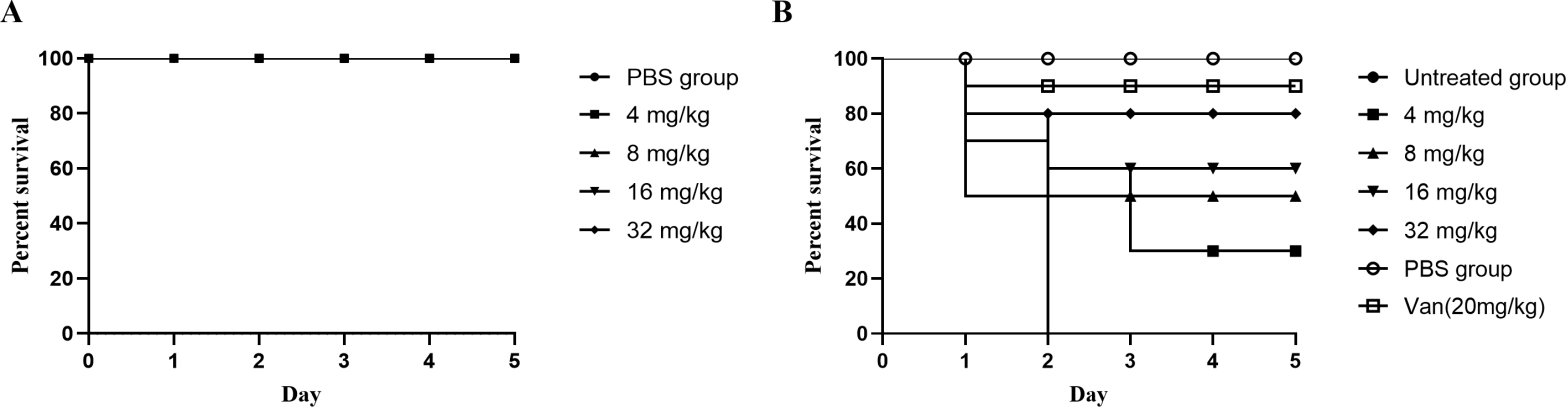
Doxifluridine showed efficacy in a *G. mellonella* infection model. (**A**) Effect of different doses of doxifluridine on the survival of *G. mellonella* larvae. The dose of doxifluridine was set to 4, 8, 16, and 32 mg/kg, respectively. The survival of the larvae was observed for 5 consecutive days; (**B**) Percent survival of *G. mellonella* after infection treatment. *S. aureus* 43300-infected *G. mellonella* were treated with PBS, 20 mg/kg vancomycin, and 4, 8, 16, and 32 mg/kg doxifluridine at 1 hour post-infection.

In this paper, we also evaluated the protective effect of doxifluridine against MRSA infection in *G. mellonella* larvae. Initially, a series of escalating doses (4, 8, 16, 32 mg/kg) were tested for toxicity in *G. mellonella*. As shown in Fig. 7A, the larvae exhibited 100% survival over 5 days at all tested concentrations. Subsequently, after infecting *G. mellonella* larvae with MRSA, we treated the infected larvae with the four previously tested concentrations of doxifluridine. Following bacterial infection, the untreated group exhibited a 100% mortality rate within 2 days (Fig. 7B), while the vancomycin-treated group showed a survival rate of 90%. Compared to the untreated group, all concentrations of doxifluridine significantly increased the survival rate of the infected larvae (Fig. 7B), with the 32 mg/kg treatment group showing an 80% survival rate at 120 hours post-infection. In summary, these results indicated that doxifluridine demonstrated significant efficacy in the *G. mellonella* model.

## DISCUSSION

COPD is a leading cause of morbidity and mortality worldwide, with an increasing burden, particularly in developing countries (39). One significant challenge in managing COPD is the presence of co-infections, with MRSA being a prominent pathogen. In COPD patients, MRSA infections are associated with increased exacerbations, hospitalizations, and mortality rates (40). Management of MRSA in COPD includes appropriate antibiotic therapy, infection control measures to prevent transmission, and vaccination strategies to reduce the incidence of infections (41). However, strategies for preventing and managing MRSA infections in COPD remain a challenge and require further research and development of new treatment modalities, especially the development of novel antimicrobial agents. Here, we further validated that doxifluridine was found to have good antibacterial activity against clinically MDR *S. aureus*. Doxifluridine demonstrated significant antibacterial activity against MRSA strains isolated from COPD patients. The MIC and MBC values for doxifluridine ranged from 0.5 to 2 µg/mL and 1–4 µg/mL, respectively, indicating potent activity at relatively low concentrations. These findings align with previous reports highlighting the drug’s effectiveness against VRE and MRSA (17). Moreover, time-kill assays revealed that doxifluridine exhibited bactericidal effects within 8 hours, surpassing the efficacy of vancomycin, which required up to 10 hours for similar outcomes. This rapid bactericidal action is crucial for managing acute exacerbations in COPD patients, reducing the duration of bacterial colonization and subsequent inflammation (3). These results suggest that doxifluridine could be a potential candidate for the development of treatments against MDR *S. aureus*, including MRSA, providing new therapeutic options for controlling MRSA infections.

Biofilm formation by MRSA is a significant challenge in clinical settings, as biofilms protect bacteria from antibiotics and the host immune response, leading to persistent infections (42, 43). The study showed that doxifluridine significantly reduced biofilm mass and viability. Crystal violet staining confirmed that doxifluridine at 8 µg/mL cleared approximately 43% of mature biofilms, highlighting its potential in treating biofilm-associated infections in COPD. Moreover, a previous study has reported that 5-fluorouracil blocked quorum sensing of biofilm-embedded MRSA in mice (44). And doxifluridine is a 5-FU prodrug. The quorum-sensing system is crucial for biofilm development and maintenance, and its disruption can significantly impair the ability of MRSA to form and sustain biofilms (45). Both doxifluridine and 5-FU showed promising anti-biofilm activity, but their mechanisms differ. Doxifluridine acted primarily by killing bacteria and reducing biofilm mass directly, while 5-FU interfered with the communication pathways (quorum sensing) that regulated biofilm formation. This distinction was significant because it suggested that doxifluridine might be more effective in rapidly reducing existing biofilm burden, whereas 5-FU could be more effective in preventing biofilm formation and maintenance. This indicated to some extent that even after metabolic transformation *in vivo*, doxifluridine could still act on the bacterial biofilm. Furthermore, doxifluridine has been shown to reduce the population of *S. aureus* persister cells. However, the extent of this reduction was relatively modest. This limited efficacy was likely attributable to doxifluridine’s mechanism of action, which depends on active DNA synthesis. Since persister cells exist in a state of low metabolic activity or dormancy, with minimal DNA replication, they are suboptimal targets for this drug (46, 47). The reduced metabolic activity and heightened resilience of persister cells may hinder doxifluridine’s ability to fully eradicate them. This underscores both the recalcitrance of persister cells and the inherent limitations of conventional antibiotics, highlighting the critical need for the development of novel therapeutics specifically targeting persister cells. In addition, one of the most promising aspects of doxifluridine was its ability to enhance the efficacy of other antibiotics. Chequerboard assays demonstrated that doxifluridine exhibited synergistic interactions with several antibiotics, reducing their MIC values by two- to eightfold. This synergy not only broadens the antibacterial spectrum but also offers a strategic advantage in managing multidrug-resistant infections by potentially lowering the required doses of each drug, thereby reducing toxicity and side effects.

Safety is a critical consideration for any therapeutic agent, particularly in vulnerable populations like COPD patients. The study’s safety evaluations revealed that doxifluridine did not exhibit hemolytic toxicity or significant cytotoxicity against mammalian cells. Furthermore, *in vivo* studies using the *G. mellonella* larvae model showed that doxifluridine significantly increased the survival rate of MRSA-infected larvae without causing toxicity at effective doses. Drug repurposing, the strategy of finding new therapeutic uses for existing drugs, offers a cost-effective and time-saving alternative to traditional drug development (48, 49). Doxifluridine, originally developed as a chemotherapeutic agent, has an established safety profile and extensive clinical usage data (50, 51). Repurposing it for treating MRSA infections in COPD patients leverages its known pharmacokinetics and toxicology, accelerating the pathway to clinical application. The study of doxifluridine in this context underscores the potential of repurposed drugs in addressing urgent public health needs. Similar approaches have been successfully applied to other drugs, such as metformin (14) and chloroquine (15), broadening their therapeutic scope beyond their original indications.

The findings from this study suggest that doxifluridine could be integrated into the treatment regimens for COPD patients suffering from MRSA infections. Its rapid bactericidal action, effectiveness against biofilms, and synergistic potential with other antibiotics make it a valuable addition to the antimicrobial arsenal. Clinical trials are warranted to further validate these results and establish optimal dosing strategies for COPD patients. Additionally, the study’s approach can serve as a model for investigating other repurposed drugs against antibiotic-resistant infections. By expanding the therapeutic options available for managing COPD exacerbations, healthcare providers can better address the challenges posed by multidrug-resistant pathogens.

## Data Availability

The data (Dataset S1) presented in this study are available on request from the corresponding author.

## SUPPLEMENTAL MATERIAL

Dataset S1 spectrum.01805-24-s0001.xlsx The data presented in this study.

ASM does not own the copyrights to Supplemental Material that may be linked to, or accessed through, an article. The authors have granted ASM a non-exclusive, world-wide license to publish the Supplemental Material files. Please contact the corresponding author directly for reuse.
