# Erratum for Zhang et al., “Doxifluridine effectively kills antibiotic-resistant *Staphylococcus aureus* in chronic obstructive pulmonary disease”

**DOI:** 10.1128/spectrum.03190-24

**Published:** 2026-03-23

**Authors:** Liansheng Zhang, Yingzhang Zhang, Lijie Tian, Qiang Shen, Xiaolong Ma

## ERRATUM

Volume 12, no. 12, e01805-24, 2024, https://doi.org/10.1128/spectrum.01805-24. Page 1, byline: Liansheng Zhang’s name should appear as shown in this Erratum.

